# Supplementary material for: Prediction of post transarterial chemoembolization MR images of hepatocellular carcinoma using spatio-temporal graph convolutional networks
Source: PLoS One. 2021 Dec 7;16(12):e0259692. doi: 10.1371/journal.pone.0259692 (PMC8651128; doi:10.1371/journal.pone.0259692)
Supplement: Attachment — Submitted filename: Rebuttal.pdf [file pone.0259692.s001.pdf]

Prediction of post transarterial chemoembolization MR images of hepatocellular carcinoma using spatio-temporal graph convolutional networks
–
PONE-D-21-29673

First and foremost, we would like to express our sincere gratitude to the editors and reviewers, for having taken the time and effort to address comments, suggestions and questions with regards to manuscript, and improve its scientific quality. We addressed all the comments and questions raised by the reviewers and enclose the following point-by point response to those issues.

Sincerely,
The Authors

Reviewer 1:

1. The authors should mention the limitation of this approach in the discussion.
> We thank the reviewer for this suggestion. We have added the following paragraph:

"The study has however some limitations. The first relates to the size of the training dataset used to create the generative model from patients treated with specific regimens of TACE. The dataset does not include the wide range of possible drug regimens and compounds, which can greatly affect the outcomes of patients, depending on tumor vascularity and viability. Even though the clinical dataset tends to be homogenous, thus helping in the model convergence from the DCE-MRI examinations pre- and post-therapy, using a more heterogenous set of patient datasets treated with different drug combinations and variation of contrast timing can help in the generalization of the model. The second issue lies in the quality of the maps used for ground-truth, which are prone to user variability. It should be noted that even though lesion maps and segmentations can be edited by radiologists, the framework is limited in incorporating variants in regimen composition or treatment course, which could be applied to each case. A final limitation lies in the model dependence on constrained timings in follow-up examinations, which can be difficult to respect for each patient, varying within 1-2 months. This makes predictions less reliable when the examinations are outside the recommended follow-up visits."

2. It's unclear how the authors selectively enroll the HCC patients in this study. It's necessary to include a figure/table to clarify this point.
> We have added a new flowchart figure (Fig. 5) describing the patient selection workflow.

3. The authors need to mention the method they used for the statistical analysis and include the significant symbol in the Tables.
> We have added a *Statistical analysis* sub-section at the end of the Methods section, detailing the Wilcoxon test (with $p<0.05$ indicating statistically significant differences) was used for

paired statistical analysis. We added information with regards to software which was used. Finally, statistically significant differences were highlighted in bold in the tables.

4. The figure legends should be arrange in better order and not between texts.
> This has been corrected in the revised manuscript. Figure legends are now at the end of paragraphs citing the figure, without cutting text.
